# Implementing EEG hyperscanning setups

**DOI:** 10.1016/j.mex.2019.02.021

**Published:** 2019-02-26

**Authors:** Paulo Barraza, Guillaume Dumas, Huanhuan Liu, Gabriel Blanco-Gomez, Marion I. van den Heuvel, Martijn Baart, Alejandro Pérez

**Affiliations:** a Centro de Investigación Avanzada en Educación (CIAE), Universidad de Chile, Santiago de Chile, Chile; b Human Genetics and Cognitive Functions Unit, Institut Pasteur, Paris, France; c CNRS UMR 3571 Genes, Synapses and Cognition, Institut Pasteur, Paris, France; d Human Genetics and Cognitive Functions, University Paris Diderot, Sorbonne Paris Cité, Paris, France; e Research Center of Brain and Cognitive Neuroscience, Liaoning Normal University, Dalian, China; f Beijing Key Laboratory of Applied Experimental Psychology, Faculty of Psychology, Beijing Normal University, Beijing, China; g Centre for French & Linguistics, University of Toronto Scarborough, Toronto, Canada; h Department of Cognitive Neuropsychology, Tilburg University, Tilburg, the Netherlands; i BCBL, Basque Center on Cognition, Brain and Language, Donostia, Spain; j Psychology Department, University of Toronto Scarborough, Toronto, Canada

**Keywords:** EEG hyperscanning setup, EEG, Hyperscanning, Interbrain, Brain-to-brain, B2B

## Abstract

Hyperscanning refers to obtaining simultaneous neural recordings from more than one person (Montage et al., 2002 [1]), that can be used to study interactive situations. In particular, hyperscanning with Electroencephalography (EEG) is becoming increasingly popular since it allows researchers to explore the interactive brain with a high temporal resolution. Notably, there is a 40-year gap between the first instance that simultaneous measurement of EEG activity was mentioned in the literature (Duane and Behrendt, 1965 [2]), and the first actual description of an EEG hyperscanning setup being implemented (Babiloni et al., 2006 [3]). To date, specific EEG hyperscanning devices have not yet been developed and EEG hyperscanning setups are not usually described with sufficient detail to be easily reproduced. Here, we offer a step-by-step description of solutions to many of these technological challenges. Specifically, we describe and provide customized implementations of EEG hyperscanning setups using hardware and software from different companies: Brain Products, ANT, EGI, and BioSemi.

• Necessary details to set up a functioning EEG hyperscanning protocol are provided.
• The setups allow independent measures and measures of synchronization between the signals of two different brains.
• Individual electrical Ground and Reference is obtained in all discussed systems.

## Graphical abstract

**Figure:**
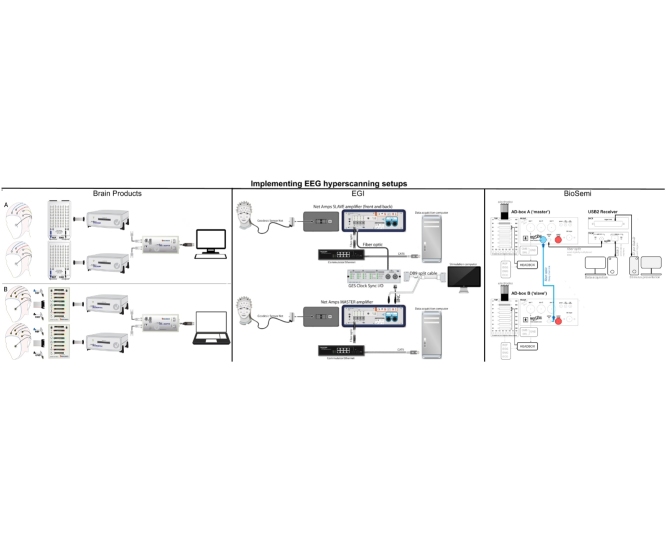

**Specifications Table**

**Table:** 

**Subject Area:**	*Psychology*
**More specific subject area:**	*Electrophysiology*
**Method name:**	*EEG hyperscanning setup*
**Name and reference of original method:**	*Article title: Differential brain-to-brain entrainment while speaking and listening in native and foreign languages*
	*Reference: https://doi.org/10.1016/j.cortex.2018.11.026*
	*Journal title: Cortex*
	*Corresponding author: Dr. Alejandro Pérez*
	*First author: Dr. Alejandro Pérez*

## Method details

### Brain products and EasyCAP

Hyperscanning with Brain Products can be implemented in two ways: A) with passive electrodes and BrainAmp standard amplifiers, and B) with active electrodes and BrainAmp DC amplifiers. Perfect synchronization between the two data-sets is achieved by recording all data in a single software workspace.

#### Necessary hardware (32-channel measurement per participant)

*Version A: Passive electrodes and BrainAmp standard amplifiers*

• EasyCAP standard recording cap. 2 units
• Set of 32 Ag/AgCl ring electrodes. 2 units
• 64-channel Electrode Input Box EIB64-A or EIB64. 2 units
• Flat ribbon cable. 2 units
• BrainAmp Standard amplifier. 2 units
• PowerPack rechargeable battery. 1 unit
• Fiber optic cable. 2 units
• USB2 Adapter, BUA64 (includes USB cable). 1 unit
• Recording computer. 1 unit
• Stimulation Computer. 1 unit
• TTL trigger cable. 1 unit

*Version B: active electrodes and BrainAmp DC amplifiers*

• ActiCHamp standard caps. 2 units
• ActiCAP 32 channels electrode set. 2 units
• ActiCAP ControlBox. 2 units
• Flat ribbon cable. 2 units
• BrainAmp DC amplifier. 2 units
• PowerPack rechargeable battery. 1 unit
• Fiber optic cable. 2 units
• USB2 Adapter, BUA64 (including cable). 1 unit
• Recording computer. 1 unit
• Stimulation Computer. 1 unit
• LPT cable. 1 unit
• TriggerBox. 1 unit

**Fig. 1:**
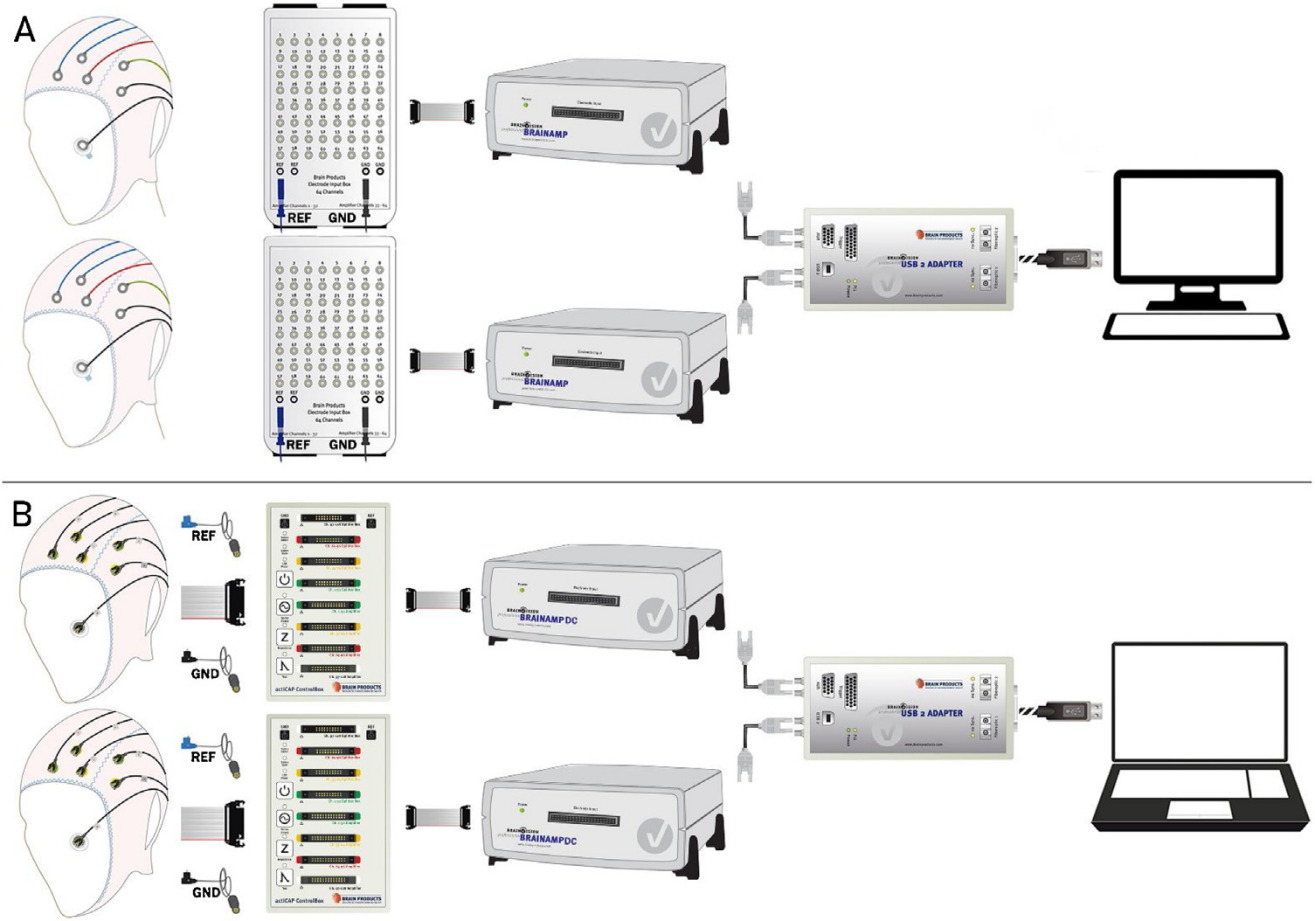
A depiction of elements and connections in the EEG hyperscanning setup using equipment from Brain Products and EasyCAP. Panel A corresponds to Version A with BrainAmp standard amplifiers. Panel B corresponds to Version B with active electrodes and BrainAmp DC amplifiers. The most important step is to connect each participant to the amplifier through individual electrode boxes (EIB64 or ControlBox, depending on the setup). This allows for individual Reference and Ground, and therefore independent recordings. Amplifiers are connected to the computer via the same USB2 adaptor. Signals from the two participants are synchronously recorded in a 64-channel workspace of the Brain BrainVision Recorder software.

Fig. 1 shows the EEG hyperscanning setup corresponding to the Brain Products systems. Panel A shows the setup with passive electrodes and BrainAmp standard amplifiers [4]. Each EasyCAP standard recording cap, mounted with up to 32 Ag/AgCl ring electrodes (placed according to the researcher’s criteria) is connected to one Electrode Input Box (see Hint 1). Importantly, Reference (REF) and Ground (GND) electrodes from each participant are connected to their individual Electrode Input Boxes EIB64-A or EIB64 (see Hint 2), that are connected to the amplifiers with flat ribbon cables. Note that both 32-channel amplifiers are fed by a single PowerPack rechargeable battery (not included in the figure for simplification). Next, each amplifier connects via a fiber optic cable to the USB2 Adapter (BUA64). Finally, the USB2 Adapter is connected to the recording PC. Note that the USB2 Adapter BUA64 is not only the USB interface between amplifiers and the recording PC but also the interface between stimulation and recording PCs. Because of this, a TTL trigger cable from the stimulation computer is also connected to the BUA64 (these elements are not included in Fig. 1 for simplification). In the same way that the two BrainAmps with 32-channels were synchronized by the USB 2 Adapter BUA64, four BrainAmps could also be synchronized by using a USB 2 Adapter BUA128 or a dualBUA solution. This simple variation will allow 64-channel recordings for each subject.

**Fig. 2:**
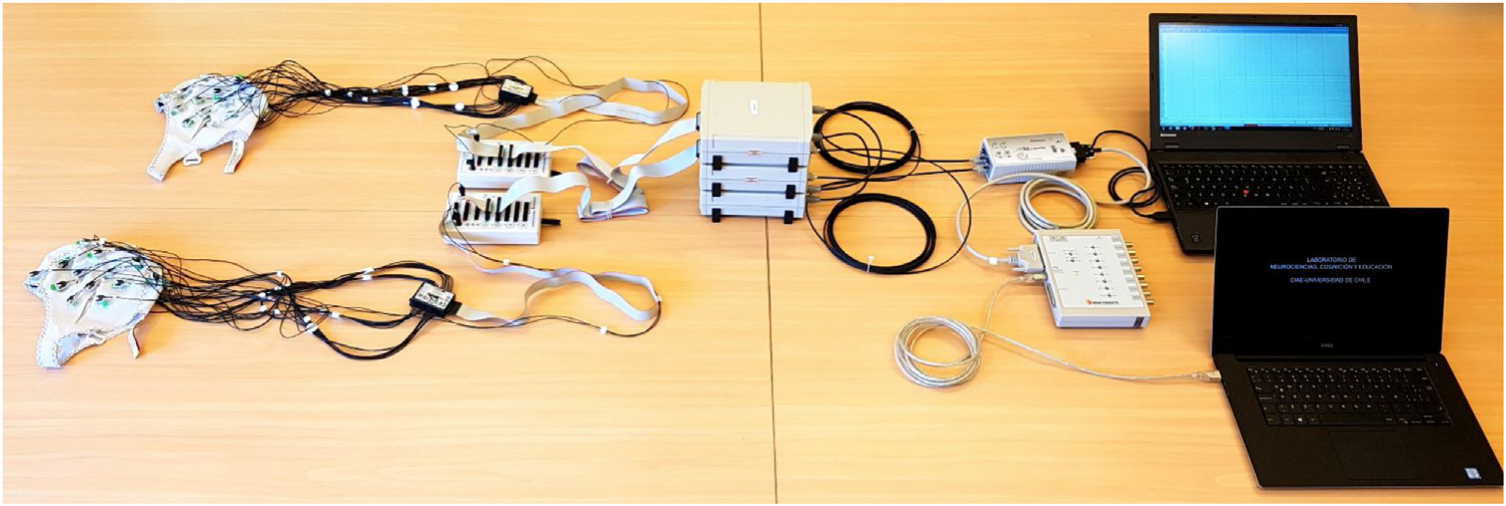
A panoramic picture showing all elements and connections described in the Version B setup, including the stimulation computer.

Fig. 1, Panel B shows the setup of the Brain Products system with active electrodes and BrainAmp DC amplifiers. Each ActiCHamp standard cap mounted with an ActiCAP 32-channel electrode set is connected to one ActiCAP ControlBox. Similar to *Version A*, each participant’s REF and GND electrodes are connected to an individual ControlBox, which is connected to one BrainAmp DC amplifier using a flat ribbon cable. The amplifiers send the EEG signal to the USB2 Adapter BUA64 via fiber optic cables, which digitize the data and send it to the recording computer via a USB cable. Here, the triggers/markers are sent from the stimulation computer to a TriggerBox via USB cable and then forwarded from the TriggerBox to the BUA via an LPT cable. See Fig. 2 for a panoramic view of the setup *Version B* clarifying this point.

#### Hints

##### Hint 1

**Fig. 3:**
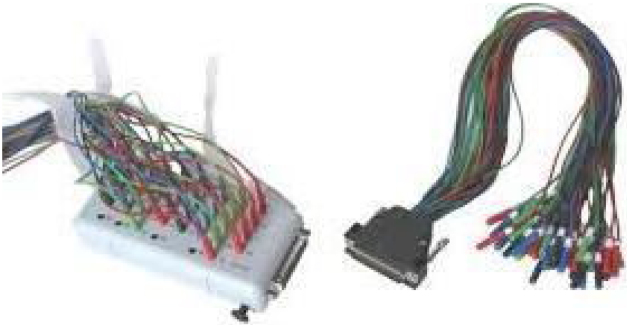
Image of the Inputbox (KI) on the left and an electrode board adaptor (KV) on the right.

**Fig. 4:**
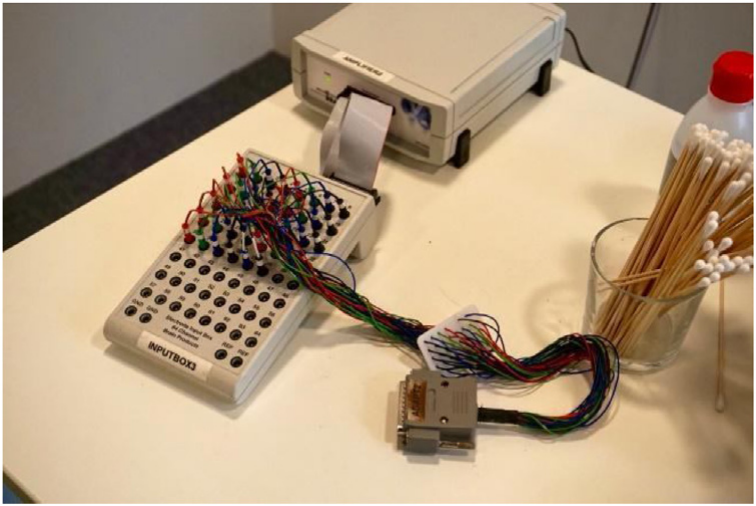
Image showing the connection between the electrode board adaptor (KV) and the Electrode Input Box BUA64-A.

For convenience, the junction of the Inputbox (KI) and electrode board adaptor (KV) devices (see Fig. 3) can bypass connections from the electrode ends to the Electrode Input Box. Using KI and KV as an intermediary step simplifies the process of connecting and detaching electrodes. Instead of directly connecting the passive electrodes to the Electrode Input Box, they are connected to the Inputbox. Consequently, the 38-pin-Dsub-connector of the Inputbox connects to the Dsub-connector of the electrode board adaptor. Lastly (see Fig. 4 for this step), the 38 single safety sockets of the electrode board adaptor connect to the Electrode Input Box.

##### Hint 2

The Electrode Input Box models EIB64 and EIB64-A can both be used as an interface between the electrodes and the amplifier. Note that the Electrode Input Box contains 64 single-input safety sockets. Either set of input sockets (1–32 or 33–64) can be used individually, but combining across the sets is not possible. The chosen socket (1–32 or 33–64) is the common connector input terminal of the EIB64 that connected to the amplifier through a flat ribbon cable.

#### Recording data with BrainVision recorder software (versions A and B)

Data is recorded using custom workspaces in the BrainVision Recorder software. Workspaces contain user-defined recording settings, such as amplifier parameters, cap configuration, electrode positions and channel names. The three different workspaces needed are provided in the Supplementary Materials 1, 2 and 3. First, impedance measurements should be checked by two individual 32-channels workspaces corresponding to each participant/amplifier. For organization purposes, we named one participant as Subject A (e.g. left participant while facing the screen) and the other as Subject B. Finally, data from both participants are recorded in a 64-channel workspace, where the first 32 channels belong to the amplifier connected to the USB2 Adapter’s port labelled Fiberoptic 1 and the second half belong to the amplifier connected to the port labelled Fiberoptic 2.

### ANT

Hyperscanning with ANT systems simply consists of two independent EEG recordings, receiving a simultaneous marker from the stimulation computer via a y-cable. Synchronization of the different data sets should be performed off-line.

#### Necessary hardware (64-channel measurement per participant)

• Waveguard original cap (64 electrodes). 2 units
• eego™mylab ANTneuro amplifier. 2 units
• USB2 cable. 2 units
• Recording computer. 2 units
• Stimulation computer. 1 unit
• 25 pin serial port y-cable (female/2 male). 1 unit

**Fig. 5:**
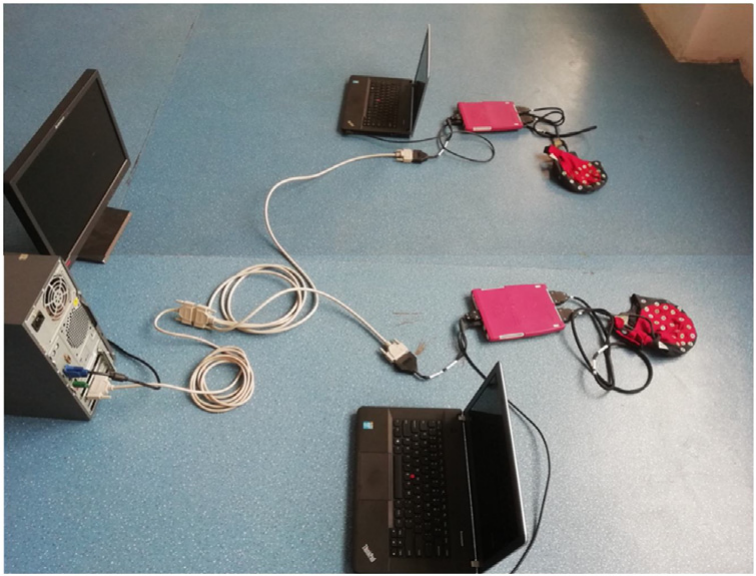
Overview of all (stimulation and recording) elements and connections in the EEG hyperscanning setup with ANT systems. Two Waveguard caps connect to two different ANT amplifiers. Each amplifier connects to an individual recording computer with USB2 cables. Critically, the stimulation computer connects simultaneously to the two ANT amplifiers by a 25-pin serial port y-cable. Triggers/markers are thus simultaneously sent to the two separate recording computers which allow synchronization of the two independent recordings at a later stage.

**Fig. 6:**
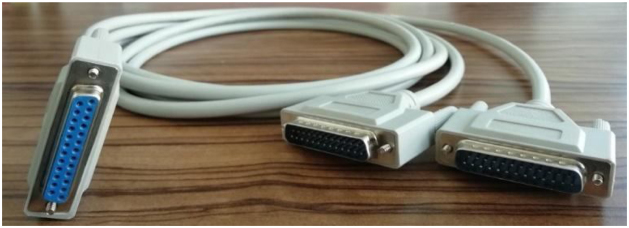
Image of a 25-pin serial port y-cable (female/2 male).

Fig. 5 shows the EEG hyperscanning setup corresponding to the ANT system. Each Waveguard cap mounted with 64 sintered Ag/AgCl electrodes is connected to an eego™mylab ANTneuro amplifier (each participant has their own independent REF and GND electrodes). The two ANT systems are connected to two different recording computers using USB2 cables. Note that there are two separate data collections running at the same time, so the timing of the triggers/markers is crucial in order to combine and synchronize the two data sets. This is facilitated by a 25-pin serial port y-cable: female/2 male (see Fig. 6) that connects one stimulation computer (with E-prime, Psychtoolbox, or other stimulation software) to both recording computers via amplifiers. The two recording computers thus simultaneously receive the same trigger/marker, which can be used to synchronize the datasets. The impedance measurements and data collection require two individual 64-channels workspaces that correspond to each participant, amplifier and computer.

### EGI

Hyperscanning setup using EGI demands the use of a clock sync box. This device coordinates the triggers sent by the stimulation computer to the two separate recordings for equal timing. The two independent recordings are later synchronized.

#### Necessary hardware (128-channel measurement per participant)

• Geodesic Sensor Net HydroCel. 2 units
• Net Amps amplifier (NA300 or NA400). 2 units
• EGI external power supply. 2 units
• Commutator Ethernet. 2 units
• CAT5 cable. 2 units
• Data acquisition computer. 2 units
• Stimulation computer. 1 unit
• BNC cable. 1 unit
• DB9 (split) cable. 1 unit
• GES Clock Sync I/O. 1 unit
• Fiber optic cable. 3 units

**Fig. 7:**
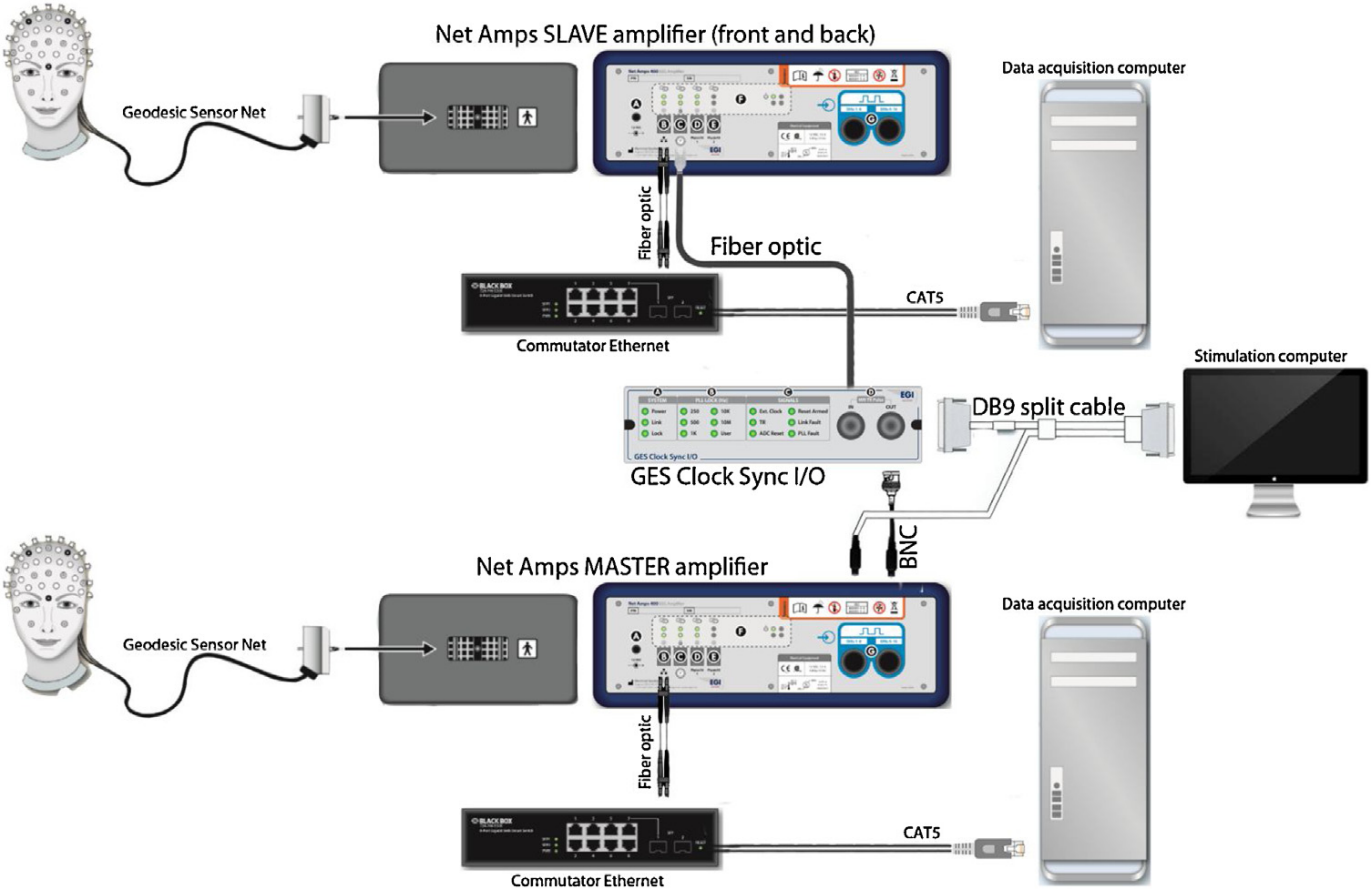
Schematic overview of the EEG hyperscanning setup using EGI systems, including stimulation and recording elements. Two HydroCel dense-array EEG Geodesic Sensor Nets connect to two different Net Amps amplifiers. Each amplifier connects to an individual EEG recording computer. The ‘master’ amplifier drives a clock sync box which then coordinates the ‘slave’ amplifier, while also dispatching the TTL triggers/markers to both amplifiers. In this way, triggers/markers are sent to the two separate recordings with equal timing, later allowing the synchronization of the two independent recordings.

Fig. 7 shows the EEG hyperscanning setup corresponding to the EGI system. The two caps with 128 channels are each connected to their own EEG Net Amps amplifier (this approach works with both 300 and 400 series). Both amplifiers are connected to a computer that runs the acquisition software, but one NetAmp is the ‘master’ and the other the ‘slave’. The ‘master’ NetAmp sends an external clock pulse through a custom BNC cable plugged into the DIN9-16 to the Clock Synchronization box. This box then sends the pulse to the clock input of the ‘slave’ NetAmp using fiber optic cables. Triggers are sent through a split DB9 cable which connects both to the DIN1-8 input of the ‘master’ NetAmp and the Clock Synchronization box. The information is sent back to the slave NetAmp through the fiber optic connection.

### BioSemi Active Two

With the BioSemi ActiveTwo system, hyperscanning is achieved via a fiber optic cable that links two AD-Boxes. This requires an input socket that is installed upon request in the BioSemi factory in Amsterdam, the Netherlands.

#### Necessary hardware (64-channel measurement per participant)

• ActiveTwo AD-Box with 7 × 8 channel Amplifiers with (retrofit) optical fiber input (‘master’ AD-box)
• ActiveTwo AD-Box with 7 × 8 channel Amplifiers (‘slave’ AD-box)
• Battery box (attached to AD-Box). 2 units
• Battery Charger. 2 units
• USB2 Receiver + cables. 1 unit
• Data acquisition computer with BioSemi ActiView recording software. 1 unit
• Stimulation computer. 1 unit
• Fiber optic cable. 2 units
• Headcaps. 2 units
• Pin-Type Active-electrodes. 4 sets (BioSemi electrode sets have 32 electrodes on a common connector)
• Flat-Type Active-electrodes for mastoid reference and/or EOG/EMG/ECG.
• Head Box. 2 units – this is optional and depends on the number of Flat-Type Active-electrodes that need to be connected and the (practical) functionality of the input sockets in the AD-Box.

**Fig. 8:**
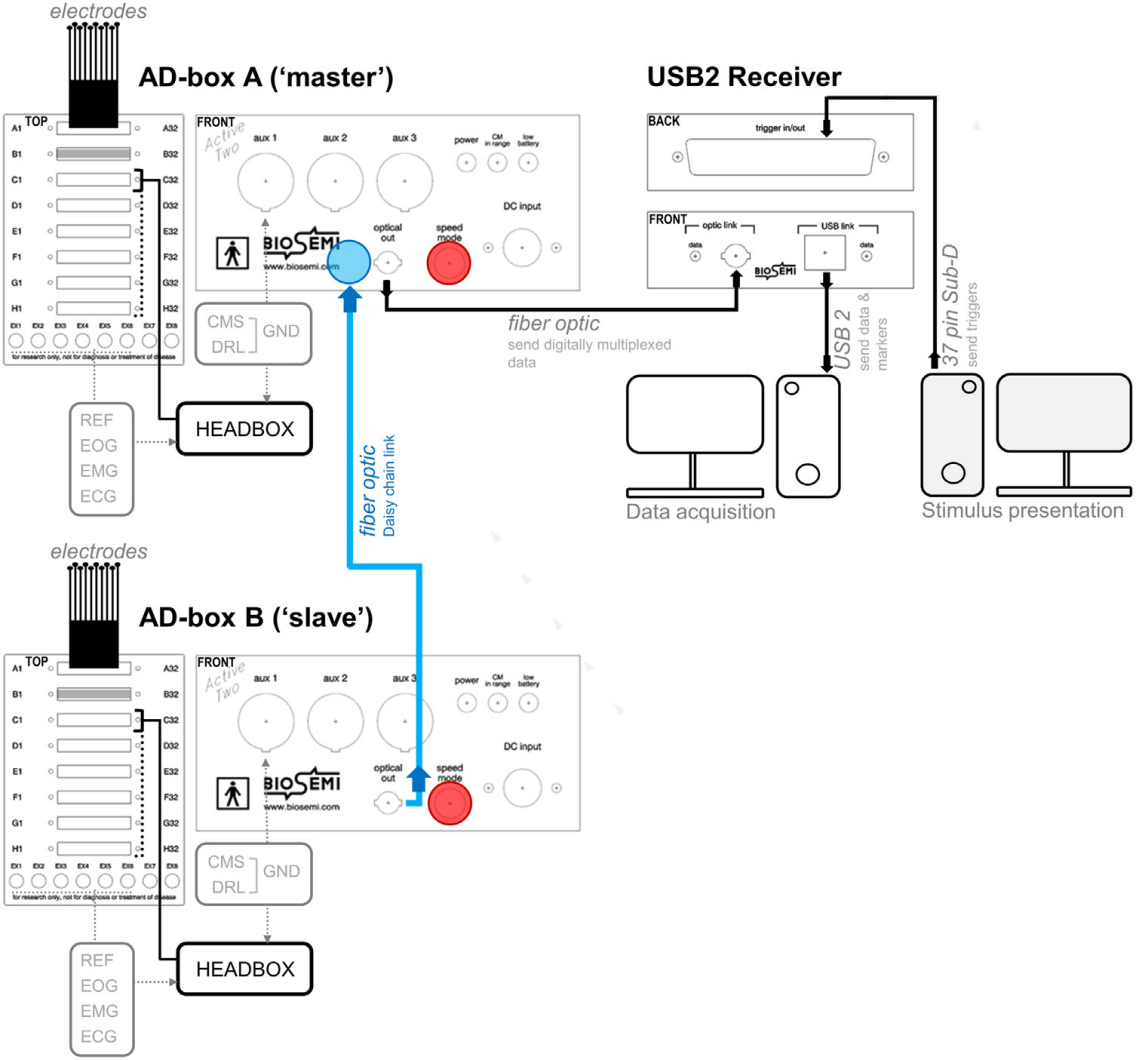
Overall schema of the EEG hyperscanning setup for the BioSemi ActiveTwo system. The data from the ‘slave’ AD-box is fed to the ‘master’ AD-box via a fiber optic cable (in blue), from which it is sent to the USB2 receiver that sends all data and trigger/marker information to the acquisition computer. Up to three ‘slave’ AD-boxes can be linked to the ‘master’ via a Daisy chain connection. The BioSemi active Ag/AgCl electrodes are combined in sets of 32 with a common connector that is fitted in the AD-Box. In the 64-channel hyperscanning set-up, 2 electrode sets per participant are thus required, that each connect to a separate input sockets in the AD-Boxes. The individual REF electrodes are Flat-Type Active-electrodes (also used for EOG/EMG/ECG) that connect either directly to the AD-Box or to a Head Box that is mounted to a free 32-pin socket on the AD-Box. The GND electrodes are ‘Pin-Type Active-electrodes’, mounted in the cap, that connect directly to the front panel of the AD-box, or to the Headbox (unless GND electrodes are integrated in the electrode band). The red circles indicate the location of the ‘speed’ setting dial which should be set to ‘0’ for the ‘master’ AD-box and to ‘1’ for the ‘slave’ AD-box.

**Fig. 9:**
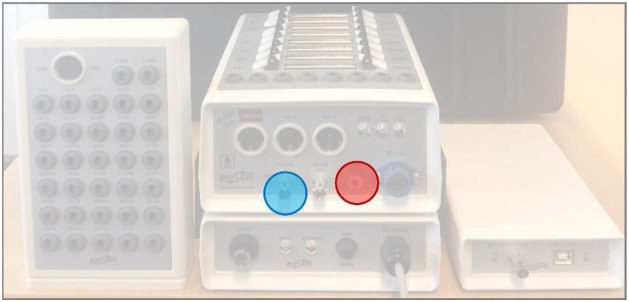
The BioSemi Headbox, the ‘master’ AD-box and battery, and the USB2 Receiver (from left to right). The blue circle indicates the retrofitted optical socket. The red circle shows where to adjust the ‘speed’ setting.

Fig. 8 shows the BioSemi ActiveTwo hyperscanning setup. Two caps with 64 electrodes (installed after cap placement) are each connected to a separate AD-Box with amplifiers. One of the two AD-Boxes is the ‘master’ system, which is connected to a USB2 Receiver, and is (Daisy chain) linked to the ‘slave’ system via a fiber optic cable (the AD-boxes need a different ‘speed’ setting: see Fig. 8, Fig. 9). This allows considerable spatial separation between participants without losing temporal alignment between the two recordings. The USB2 Receiver receives triggers/markers from the stimulus computer, and this information is combined with the data from both AD-boxes and sent to the software acquisition computer via USB2. In this implementation, the optical fiber input socket in the ‘master’ AD-box is necessary, but it is not part of the standard AD-box configuration. New systems can be ordered with this feature, and existing systems can be upgraded by BioSemi (provided that they are compatible with the retrofit). Each participant has individual REF and GND (active CMS/passive DRL loop) channels, which are connected to the individual AD-boxes. In the acquisition software (ActiView), the EEG data from both participants (and the triggers/markers) can be viewed separately, but the data is stored in one data-file with distinctive labels for each participant.

## Additional information

Brain Products setup using actiCHamp is not described since this amplifier was not explicitly designed for EEG hyperscanning. An important point to consider with the actiCHamp is that all modules share the same GND. This implies that GND has to be shared among the subjects. Also, the two subjects cannot have a dedicated on-line REF with this system. Therefore, the EEG activity during the recording will look different from single-subject recordings, because it will be affected by the different values of the GND from the two subjects. We consider the shared GND as a possible confounding factor (i.e. no-go) at least for studies trying to classify ‘real’ interacting dyads from artificially created ones. However, dual recordings have been successfully conducted with actiCHamp amplifiers by using a y-cable (GND distributor) that should be connected from the two GNDs coming from both participants into the GND port on the actiCHamp. Later (off-line), each EEG dataset (from each subject) could be separately re-referenced.
